# CyanoBase and RhizoBase: databases of manually curated annotations for cyanobacterial and rhizobial genomes

**DOI:** 10.1093/nar/gkt1145

**Published:** 2013-11-25

**Authors:** Takatomo Fujisawa, Shinobu Okamoto, Toshiaki Katayama, Mitsuteru Nakao, Hidehisa Yoshimura, Hiromi Kajiya-Kanegae, Sumiko Yamamoto, Chiyoko Yano, Yuka Yanaka, Hiroko Maita, Takakazu Kaneko, Satoshi Tabata, Yasukazu Nakamura

**Affiliations:** ^1^Center for Information Biology, National Institute of Genetics, Research Organization of Information and Systems, Yata, Mishima 411-8540, Japan, ^2^Database Center for Life Science, Research Organization of Information and Systems, 2-11-16 Yayoi, Bunkyo-ku, Tokyo 113-0032, Japan, ^3^Faculty of Life Sciences, Kyoto Sangyo University, Motoyama, Kamigamo, Kita-Ku, Kyoto 603-8555, Japan and ^4^Kazusa DNA Research Institute, 2-6-7 Kazusa-Kamatari, Kisarazu 292-0818, Japan

## Abstract

To understand newly sequenced genomes of closely related species, comprehensively curated reference genome databases are becoming increasingly important. We have extended CyanoBase (http://genome.microbedb.jp/cyanobase), a genome database for cyanobacteria, and newly developed RhizoBase (http://genome.microbedb.jp/rhizobase), a genome database for rhizobia, nitrogen-fixing bacteria associated with leguminous plants. Both databases focus on the representation and reusability of reference genome annotations, which are continuously updated by manual curation. Domain experts have extracted names, products and functions of each gene reported in the literature. To ensure effectiveness of this procedure, we developed the TogoAnnotation system offering a web-based user interface and a uniform storage of annotations for the curators of the CyanoBase and RhizoBase databases. The number of references investigated for CyanoBase increased from 2260 in our previous report to 5285, and for RhizoBase, we perused 1216 references. The results of these intensive annotations are displayed on the GeneView pages of each database. Advanced users can also retrieve this information through the representational state transfer-based web application programming interface in an automated manner.

## INTRODUCTION

Cyanobacteria constitute a large taxonomic group within the domain of eubacteria. They are widely used as model organisms to study the fundamental aspects of photosynthesis, in basic and applied plant-related research, in biotechnology for the development of third-generation biofuels and for their evolutionary contributions for the whole biosphere. CyanoBase was originally developed as a genome database for *Synechocystis* sp. PCC 6803, the first cyanobacterial genome sequenced in 1996 (1). CyanoBase subsequently has been extended to include additional cyanobacteria and related species (2–4), covering 39 organisms. Rhizobia, a collective name of the genera *Rhizobium*, *Sinorhizobium*, *Mesorhizobium* and *Bradyrhizobium*, are agronomically important bacteria because they have the ability to establish nitrogen-fixing symbioses with leguminous plants. RhizoBase was initiated as a genome database for *Mesorhizobium loti* strain MAFF303099 sequenced in 2000 (5) and was extended to include other rhizobia and related species, encompassing 18 organisms till date.

Regarding CyanoBase and RhizoBase, we have been accumulating gene annotations by incorporating evidence from published data. To maintain the quality of annotations, the involvement of the research communities of cyanobacteria and rhizobia was essential. Therefore, to assist in the submission procedure of new annotations, we developed the TogoAnnotation system (6) and also conducted in-house curation efforts to ensure that annotations are as comprehensive as possible. New sequencing technologies and automatic genome processing pipelines [e.g., MiGAP (7) and DNA Databank of Japan (DDBJ) Pipeline (8,9)] have been certainly accelerating prokaryotic genome analyses. However, it is difficult to estimate the functions of predicted genes without the information from carefully curated reference annotations of model organisms. Thus, for this, the manually curated annotations in CyanoBase and RhizoBase provide fundamental information for the interpretation of high-throughput sequencing data.

Regarding data reusability, it is important to provide a high level of accessibility and interoperability of the reference annotations. For accessibility, CyanoBase and RhizoBase use a common database system to provide the same types of functionalities, user interfaces and application programming interfaces. For interoperability, we have introduced Semantic Web technologies (10) for representing data in a standard format and providing an advanced query interface.

## DATA CURATION

### Reference genomes

CyanoBase and RhizoBase integrate reference genomes from original genome projects conducted by Kazusa DNA Research Institute and from public sequence databases. By the inclusion of recent genome sequencing projects, we added 4 and 17 new genome entries in CyanoBase and RhizoBase, respectively (4,5). As a result, CyanoBase is extended to currently include 39 completely sequenced genomes, and RhizoBase contains 18 completely sequenced genomes and two partially sequenced genomic regions, such as the symbiosis island (newly incorporated genomes are listed in Supplementary Table S1). We have integrated automatic gene annotations including BLAST and the InterPro search results in the new cyanobacterial and rhizobial genomic databases before the manual curations described in the following sections.

### Manual curation

**Figure 1.**
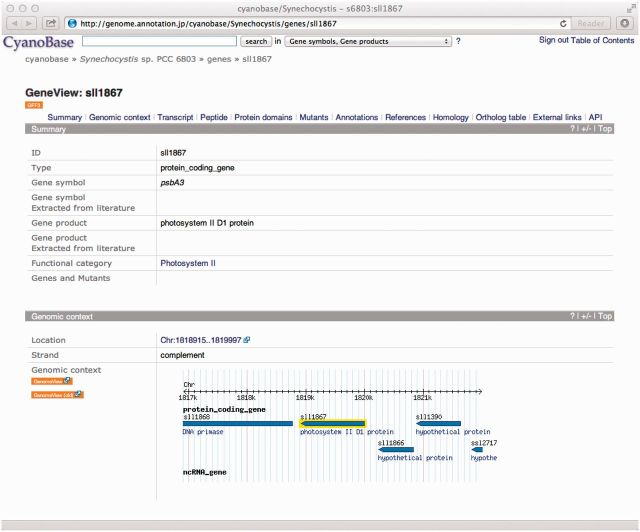
An example GeneView page for the sll1867 gene of *Synechocystis* sp. PCC 6803. Manually curated gene symbol(s) and gene product(s) are shown in the ‘Gene symbol Extracted from literature’ and ‘Gene symbol Extracted from literature’ fields in the ‘Summary’ section.

Expert curators extracted gene symbols and full names from full sections of the peer-reviewed research literature and annotated them using the Sequence Ontology (SO) terms (11) to indicate types of annotations. These annotations are immediately reflected in the ‘Extracted from literature’ fields in the ‘Summary’ section of the GeneView page of each database (Figure 1). We have been accepting community submissions to both databases including gene structure refinements, gene families, gene functions, gene symbols and links to other resources. In addition, submitted data are manually inspected by expert curators before becoming integrated.

### Curation platform

Manual curation is still one of the most important and most difficult tasks in genome projects. Therefore, methodological and technological solutions are urgently needed to reduce annotation costs. To address this issue, we have developed a web-based genome annotation tool, TogoAnnotation (http://togo.annotation.jp). This tool, which is derived from KazusaAnnotation (4), provides an easy way to access, edit and store annotation data over a flexible web interface based on social bookmarking web services architecture.

### Curated genes

CyanoBase and RhizoBase have grown considerably since their introduction. The content of CyanoBase and RhizoBase and their composition are summarized in Table 1. A statistical summary of annotations conducted in August 2013 indicated that 138 896 cyanobacterial genes were curated from 5285 published references. Hence, the number of references investigated for CyanoBase increased by 3025 in comparison with our previous report in 2010 (4). For example, of the 3725 genes contained in the *Synechocystis* sp. PCC 6803 genome, 3067 (82.3%) have been already annotated with gene symbols, protein names and gene definitions from the literature. Users are able to access the annotation of each gene on the ‘Reference’ section of the GeneView page and to find annotated data [e.g. the photosystem II D1 protein (psbA3) currently have 386 citations http://genome.microbedb.jp/cyanobase/Synechocystis/genes/sll1867#references].

**Table 1.** Number of curated publications and annotated genes for each organism of CyanoBase and RhizoBase

Database	Organism	References	Annotations	Annotated genes	Total genes
CyanoBase	*Synechocystis sp.* PCC 6803	2346	80 204	3064	3725
CyanoBase	*Anabaena sp.* PCC 7120	959	29 154	2754	6223
CyanoBase	*Synechococcus elongatus* PCC 7942	815	17 060	794	2715
CyanoBase	*Thermosynechococcus elongatus* BP-1	270	6768	2528	2528
CyanoBase	*Synechococcus sp.* PCC 7002	264	3999	265	3235
CyanoBase	*Nostoc punctiforme* ATCC 29133	151	3349	768	6794
CyanoBase	*Chlorobium tepidum* TLS	143	5532	751	2310
CyanoBase	*Anabaena variabilis* ATCC 29413	119	1731	258	5724
CyanoBase	*Prochlorococcus marinus* MED4	64	2155	390	1756
CyanoBase	*Gloeobacter violaceus* PCC 7421	52	5600	4483	4484
CyanoBase	*Prochlorococcus marinus* MIT9313	44	919	248	2326
CyanoBase	*Prochlorococcus marinus* SS120	37	539	135	1928
CyanoBase	*Arthrospira platensis* NIES-39	9	787	260	6676
CyanoBase	*Trichodesmium erythraeum* IMS101	5	22	14	4498
CyanoBase	*Synechococcus sp.* WH8102	5	38	22	2579
CyanoBase	*Synechococcus elongatus* PCC 6301	2	5	2	2580
RhizoBase	*Bradyrhizobium japonicum* USDA110	550	26 636	8366	8374
RhizoBase	*Sinorhizobium meliloti* 1021	240	9801	1990	6287
RhizoBase	*Mesorhizobium loti* MAFF303099	115	2373	865	7343
RhizoBase	*Rhizobium sp.* pNGR234ab	107	5224	989	990
RhizoBase	*Rhizobium leguminosarum* bv. viciae 3841	83	3426	781	7342
RhizoBase	*Rhizobium sp.* NGR234	8	46	17	6437

## AVAILABILITY

### Application programming interface

CyanoBase and RhizoBase are based on the same in-house developed genome database system offering a representational state transfer-based web application programming interface for automated retrieval of data by third-party tools and computer programs. As an output, various widely used formats are supported, including TSV, CSV, FASTA and GFF3 (4).

### Semantic Web application

To improve data integration within CyanoBase, RhizoBase and other microorganism databases in the near future, we have introduced Semantic Web technologies for the standard representation and common exchange protocol of data (10). First, we developed a generic ontology for semantically describing genomic annotations in cooperation with the DDBJ and the Database Center for Life Science (DBCLS). Based on this ontology, we converted annotations stored in the CyanoBase and RhizoBase databases into the resource description framework (RDF) format. The result is accessible from our SPARQL Protocol and RDF Query Language (SPARQL) endpoint at http://genome.microbedb.jp/sparql. A list of available resources is summarized in Table 2.

**Table 2.** Summary of data types and the number of items accessible from the SPARQL endpoint

Data type	Number	RDF	Reference
CyanoBase			
Genome project	39	○	
Gene	138 896	○	
Publication	5285		
Operon^a^	86	○	
Protein complex^a^	68	○	
Protein–protein interaction	3054	○	(12)
RhizoBase			
Genome project	20	○	
Gene	116 140	○	
Publication	1216		
Protein–protein interaction	2987	○	(13)

Currently, databases of bacterial model organisms are maintained and distributed independently. To ensure that these data are interoperable for a large-scale genomic analysis, we collaborated with the MicrobeDB.jp (http://microbedb.jp/) and the TogoGenome (http://togogenome.org/) projects for sharing prokaryotic genome annotations as RDF data through respective SPARQL endpoints. Such standardization reduces duplicated efforts and improves reusability while allowing each database to update their own resources independently. In addition, it is beneficial for end users that they can use a variety of data sources with common software through the standard web service interface in a unified and automated manner.

### Change of site URL

We have migrated the server hosting CyanoBase and RhizoBase from Kazusa DNA Research Institute to the National Institute of Genetics. Consequently, the location of these databases has changed to http://genome.microbedb.jp/.

### Social media

We have been delivering timely announcements on Twitter. Users can follow @cyanobase and @rhizobase on Twitter to receive the latest information on database updates and server maintenance of the CyanoBase and RhizoBase databases.

### License

All data in our database is provided under the Creative Commons CC0 public domain license (4).

## SUPPLEMENTARY DATA

Supplementary Data are available at NAR Online.

## FUNDING

Integrated Database Project, Ministry of Education, Culture, Sports, Science and Technology of Japan; National Bioscience Database Center (NBDC) of the Japan Science and Technology Agency (JST); Kazusa DNA Research Institute Foundation. Funding for Open Access: National Bioscience Database Center.

*Conflict of interest statement*. None declared.
